# Contribution of Microglia-Mediated Neuroinflammation to Retinal Degenerative Diseases

**DOI:** 10.1155/2015/673090

**Published:** 2015-03-22

**Authors:** Maria H. Madeira, Raquel Boia, Paulo F. Santos, António F. Ambrósio, Ana R. Santiago

**Affiliations:** ^1^Centre of Ophthalmology and Vision Sciences, IBILI, Faculty of Medicine, University of Coimbra, Azinhaga de Santa Comba, 3004-548 Coimbra, Portugal; ^2^Center for Neuroscience and Cell Biology, University of Coimbra, Coimbra, Portugal; ^3^Department of Life Sciences, University of Coimbra, Coimbra, Portugal; ^4^AIBILI, Coimbra, Portugal

## Abstract

Retinal degenerative diseases are major causes of vision loss and blindness worldwide and are characterized by chronic and progressive neuronal loss. One common feature of retinal degenerative diseases and brain neurodegenerative diseases is chronic neuroinflammation. There is growing evidence that retinal microglia, as in the brain, become activated in the course of retinal degenerative diseases, having a pivotal role in the initiation and propagation of the neurodegenerative process. A better understanding of the events elicited and mediated by retinal microglia will contribute to the clarification of disease etiology and might open new avenues for potential therapeutic interventions. This review aims at giving an overview of the roles of microglia-mediated neuroinflammation in major retinal degenerative diseases like glaucoma, age-related macular degeneration, and diabetic retinopathy.

## 1. Introduction

### 1.1. Retinal Structure and Cell Types

**Figure 1:**
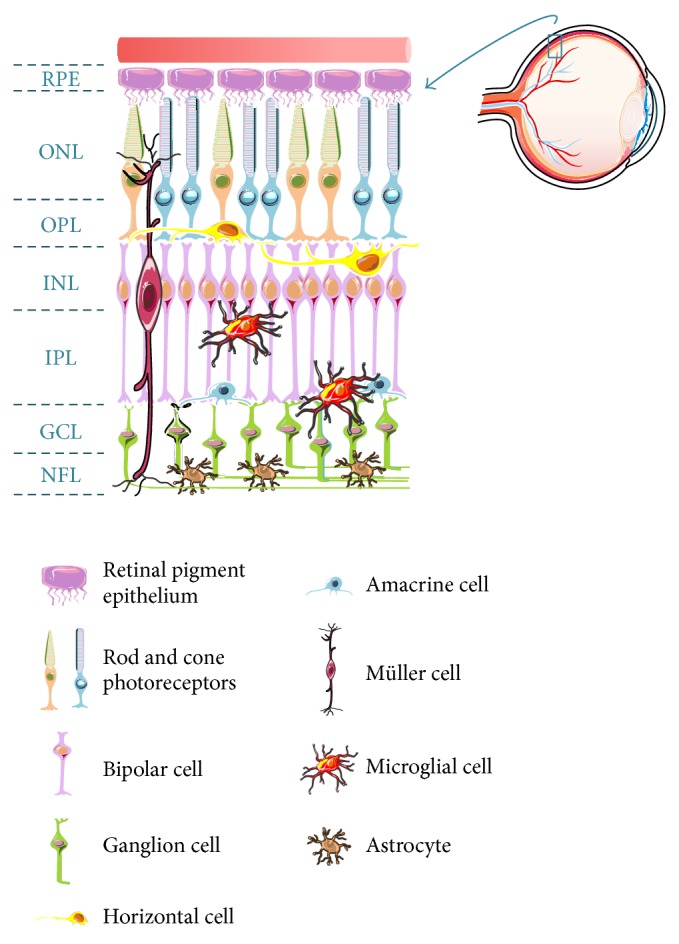
Schematic representation of the major retinal cell types and their organization in the retina. The outermost part of the retina is the retinal pigment epithelium (RPE), which consists of a monolayer of cuboid, pigmented cells between the photoreceptors and the choroid. The retina is divided into three laminar layers: the outer nuclear layer (ONL), the inner nuclear layer (INL), and the ganglion cell layer (GCL). The nuclei of rod and cone photoreceptors are located in the ONL. The INL comprises the nuclei of the bipolar, horizontal, and amacrine cells. Cell bodies of the retinal ganglion cells are present in the GCL, and their axons form the nerve fiber layer (NFL), just beneath the GCL. Synapses between photoreceptors and interneurons are located in the outer plexiform layer (OPL) and interneurons synapse with RGC in the inner plexiform layer (IPL). Müller cells span all retinal layers. Microglia are mainly found in IPL and GCL, whereas astrocytes are located near the NFL.

The retina is part of the central nervous system (CNS) due to its neuroectodermal origin and derivation from the anterior neural tube. The mature mammalian retina is structured in nuclear layers of neurons. The outermost layer of the retina is the retinal pigment epithelium (RPE), which is followed by the outer nuclear layer (ONL) that contains the cell bodies of photoreceptors. The inner nuclear layer (INL) contains the cell bodies of the bipolar, horizontal, and amacrine cells, and the ganglion cell layer (GCL) is composed by the nuclei of retinal ganglion cells (RGCs) and of displaced amacrine cells. These cells are interconnected through synapses that occur in the outer and inner plexiform layers (Figure 1). Besides neurons, other cells are present in the retina, such as glial cells (Müller cells, astrocytes, and microglia) and the cells that constitute the retinal vessels (endothelial cells and pericytes). The RPE is a monolayer of cuboid, pigmented cells in which the apical membrane faces the photoreceptor outer segments, with important functions for retinal physiology (reviewed in [1]).

Photoreceptors transduce light energy into electrochemical signals to the second-order neurons, bipolar cells, which synapse with RGCs (vertical pathway). Amacrine and horizontal cells modulate this pathway of information, commonly referred to as the horizontal visual pathway. The axons of RGCs form the optic nerve and extend to the lateral geniculate nucleus (LGN) in the thalamus and the superior colliculus in the midbrain, from which information is further transmitted to the visual processing centers in the visual cortex [2, 3].

Müller cells constitute the predominant glia in the vertebrate retina, spanning the entire thickness of the retina. These cells are responsible for the homeostatic and metabolic support of retinal neurons and are involved in the regulation of the synaptic activity in the inner retina [4–6], but they also contribute to increase photon absorption by cones [7].

Astrocytes, which have flattened cell bodies and fibrous radiating processes, enter the developing retina from the brain along the developing optic nerve, exerting an important role on structural support of the retina. Together with Müller cells, astrocytes integrate the vascular and neuronal activity of the retina [6, 8].

The third type of glial cells is present in the retina is microglia, the tissue-resident immune cells, which are constantly surveying the parenchyma (reviewed in [9]). Microglial cells are crucial effectors and regulators of changes in homeostasis during development and in health and disease. Although the functions of retinal microglia under physiological conditions are not extensively clarified, the importance of the interactions between microglia and both neurons and macroglia to the homeostasis of the retina is strongly recognized. Microglial cells are implicated in many functions essential for the proper development of the CNS, from neurogenesis to synaptic pruning, the process of synapse elimination (reviewed in [10, 11]). In the retina, TGF-*β* may have a role in regulating microglia-mediated synaptic pruning [12, 13]. Microglial cells are also involved in programmed cell death in the developing retina, and nerve growth factor released by microglia may induce retinal neuronal cell death [14]. Microglial cells interact with neurons in a reciprocal form, by balancing excitatory and inhibitory neurotransmission, which contributes to the maintenance of neuronal activity and microglia homeostasis in the healthy brain (reviewed in [15]). Neurotrophic factors released by microglia have an impact on neuronal physiology and survival. Brain derived neurotrophic factor (BDNF), ciliary neurotrophic factor (CNTF), glial cell line-derived neurotrophic factor (GDNF), NGF, neurotrophin-3 (NT3), and basic fibroblast growth factor (bFGF) have been shown to protect and regulate the survival of photoreceptors [16]. Microglial cells also establish important interactions with Müller cells, regulating the microglia-Müller-photoreceptor network that serves as a trophic factor-controlling system during retinal degeneration [17]. The bidirectional communication between microglia and Müller cells have been suggested to act as a mediator of neuron-microglia interaction, acting as a sensor of neurotransmission signals resulting from neuronal and synaptic activity [4, 18–20]. Also, Müller cells may provide ATP to the extracellular environment that mediates the activity-dependent regulation of microglial dynamic process motility [19].

## 2. Origin of Retinal Microglia

Microglial cells were first described by Pio del Rio-Hortega in 1932, as a unique cell type that differs from other glial and neuronal cells in morphology and constitutes approximately 5% to 12% of the cells of the CNS (reviewed in [21]).

Microglial cells are from mesodermal/mesenchymal origin deriving from myeloid progenitors that migrated from the periphery during late embryonic and postnatal life (reviewed in [9, 22, 23]). Taking into account the similarities between microglia and peripheral macrophages, it is reasonable to understand the major challenge that researchers have been facing to distinguish these two cell types. Nevertheless, recent evidence provided by gene expression profile studies suggest that microglia differs considerably from macrophages allowing the identification of unique molecular signatures [24, 25].

Concerning the retina, the precursors of microglia emerge during retinal development, prior to vascularization, via the ciliary margin, and differentiate in ramified, quiescent parenchymal microglia in the adult retina [26]. Radiation bone marrow chimeras have been used to assess microglia turnover and replenishment. Using these models, retinal microglia turnover was reported to take six months in the mouse [27, 28] and one year in rats [27, 28]. However, the use of parabiotic mouse model, which obviates the need for irradiation and bone marrow transfer, provided the evidence that under physiological conditions there is no turnover of microglia [29]. Most probably, microglia turnover observed in radiation chimeras is due to irradiation treatment that can act as an insult to the eye, stimulating the turnover of cells derived from bone marrow in ocular tissues [30]. These findings suggest that maintenance and local expansion of microglia are solely dependent on the self-renewal of resident cells.

In the developing retina, microglial cells have been found to be crucial for retinal growth and neurogenesis [31]. Additionally, undifferentiated microglial cells have also been associated with increased production of nitric oxide (NO) [32] and promotion of neuronal cell engulfment during retinal development [33].

In the adult retina, microglial cells are distributed in the plexiform layers, GCL and nerve fiber layer (NFL), showing highly motile protrusions that survey the surrounding environment [26, 34–39]. The movement of their processes occurs in all directions, and it is unaccompanied by soma migration [40], suggesting that the process dynamics may also serve to exchange signals between neighboring microglia, and may help explaining laminar retinal microglia distribution [41]. Interestingly, in the adult retina, microglial cells have different morphologies throughout the different layers. In the NFL, microglial cells are scarce and have a bipolar morphology, with long axis parallel to the course of RGC axons. Multipolar microglial cells, with round or oval cell bodies and some main processes, can be found in the GCL. Microglial cells in the IPL have small round cell bodies with three main branches that are stratified and distributed through the entire retina [42].

The functions of microglia in the physiology of the retina are not fully elucidated yet. Nevertheless, microglial cells are required for normal retinal growth and neurogenesis [31] and proper retinal blood vessel formation [43].

## 3. Microglia Responses in Neurodegenerative Disorders

Neurodegeneration describes the slow and progressive dysfunction and loss of neurons or their axons in the CNS. Despite different triggering events, microglia activation is a major characteristic of neurodegenerative conditions, such as Alzheimer's and Parkinson's diseases, traumatic brain injury, and multiple sclerosis (reviewed in [44–47]). Contrary to what happens in conditions of acute inflammation, where microglia activation may have beneficial effects (elimination of pathogens and cell debris), in chronic neuroinflammation microglia activation is usually detrimental, contributing to the pathogenesis of neurodegenerative disorders (reviewed in [22]). Chronic neuroinflammation encompasses overactivated microglial cells releasing proinflammatory mediators and increased oxidative and nitrosative stress [48]. Activated microglial cells can proliferate and migrate to the site of injury, where the morphological alterations are usually accompanied by changes in signaling and gene expression [49]. Usually, upon injury, the levels of CD45 are elevated, making it difficult to distinguish microglia from infiltrating macrophages [50]. Exacerbated and sustained neuroinflammation creates a toxic milieu that may lead to detrimental effects in neuronal cells [46, 51].

## 4. Retinal Degenerative Diseases and Neuroinflammation

Nearly 285 million of people have visual impairment, and this number is expected to continue to increase as a result of the ageing of the world's population [52].

**Figure 2:**
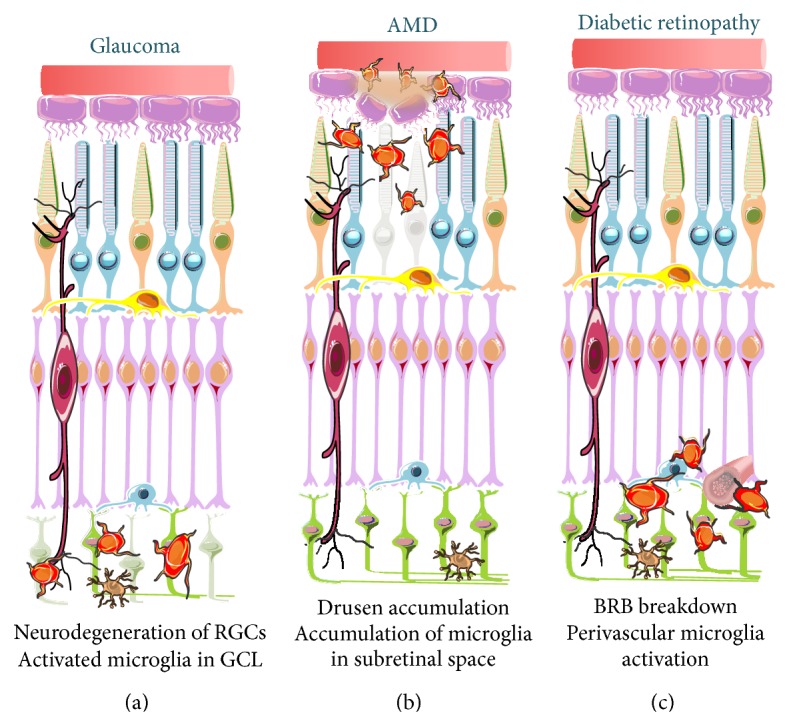
Schematic representation of microglial responses in glaucoma, AMD, and diabetic retinopathy. Reactive microglial cells are found in the retina of patients and animal models of glaucoma (a), AMD (b), and diabetic retinopathy (c). (a) Glaucomatous retinas present abnormally distributed activated microglial cells in the GCL, namely, surrounding the RGC axons and soma. (b) In AMD retinas, microglial cells accumulate in the ONL and subretinal space, surrounding drusen deposits. (c) Increased vascular permeability in diabetic retinopathy is accompanied by perivascular accumulation of activated microglial cells.

Retinal degenerative diseases, such as glaucoma, age-related macular degeneration (AMD), and diabetic retinopathy, are among the main causes of blindness worldwide [53]. These retinal diseases are characterized by chronic neuroinflammation and microglial cells have a key role in the initiation and perpetuation of the inflammatory response (Figure 2). The overactivation of microglia results in excessive production of inflammatory mediators that accumulate to levels that are harmful to neurons, further contributing to retinal neurodegeneration [54, 55].

### 4.1. Glaucoma

Glaucoma affects approximately 70 million people worldwide and nearly 2% of the population over the age of 40 [56, 57]. Glaucoma is defined as a group of disorders characterized by optic neuropathy with clinically visible alterations at the ONH encompassing thinning of the neuroretinal rim and excavation of the optic disc and representing progressive loss of RGCs and their axons [58].

Several factors are associated with the development and progression of glaucoma, such as family history, systemic hypertension, diabetes, and cigarette smoking, but the main risk factors are elevated intraocular pressure (IOP), above 21.5 mmHg, and age [59]. Current therapeutic approach is focused on lowering IOP by pharmacological means, surgically or with laser treatment. However, despite efficient IOP control, a vast majority of patients continue to lose vision [60], emphasizing the need of alternative drug-based neuroprotective treatments that target RGC apoptosis and inflammatory pathways [61–63].

Neuroinflammation has been recognized as playing an important role in the pathogenesis of glaucoma. Increased levels of inflammatory mediators, such as tumor necrosis factor (TNF) [64–69], interleukin (IL)-6 [69–74], IL-9, IL-10, IL-12 [75], and NO [76, 77], are found in the retina and aqueous humor of patients and in experimental glaucoma.

TNF has been implicated as a mediator of RGC death in glaucomatous retina [66, 78–80]. Production and release of TNF increase following elevated IOP or ischemia, suggesting TNF as an attractive therapeutic target. Indeed, RGC apoptosis is attenuated by a neutralizing antibody against TNF [81]. Moreover, etanercept (Enbrel), a widely used TNF antagonist, attenuates inflammation and RGC loss in a glaucoma animal model [82]. Recently, it was reported that polymorphisms in IL-1*β* gene might contribute to the increased risk of primary open angle glaucoma but not to the progression [83]. Inducible nitric oxide synthase (iNOS) is usually upregulated by inflammatory mediators producing large amounts of NO [84, 85]. Upregulated iNOS and increased NO levels were found in the ONH of glaucomatous patients [86] and in the retina and ONH of glaucoma animal models [77, 87, 88]. Inhibition of iNOS with aminoguanidine confers neuroprotection to RGCs in an animal model of glaucoma [89], supporting a role of NO in the pathophysiology of glaucoma.

IL-6 has been proposed has a key component of pressure-induced responses by retinal microglia [90, 91]. In genetic animal models of glaucoma alterations in the expression of IL-6 and IL-6 receptors have been detected [74]. Similarly, in the aqueous humor of patients with neovascular glaucoma, the levels of IL-6 increase spatial and temporarily correlated with the grade of neovascularization of the patient [70]. Nevertheless, IL-6 increases the survival of RGCs challenged with pressure, suggesting that it may be an attempt to regenerate RGC axons [73, 91].

Specific changes in autoantibody profiles have been described in glaucomatous patients and animal models [92–94], which are associated with antibody depositions in the sera and aqueous humor of glaucomatous patients, and increased microglial cell activation in the retina of experimental models [95]. These changes have been linked with the inflammatory process that precedes RGC degeneration and clearance of cell debris [96].

Microglial cells are considered to have a key role in the inflammatory environment in glaucomatous conditions. Several studies focusing on the role of microglial cells in glaucoma have shown that these cells have alterations in morphology, gene expression, cell proliferation, cell adhesion, and immune response, compatible with a reactive phenotype [89, 97–101]. In fact, growing evidence demonstrates that the interactions between RGCs and glia are critically important for glaucomatous neurodegeneration [98, 102–104].

Abnormal microglia reactivity and distribution have been observed in animal models of RGC degeneration, as the optic nerve axotomy model [105–107] and retinal ischemia [77, 108], suggesting that microglia become reactive secondary to RGC degeneration. Nevertheless, direct evidence of the contribution of microglia to the loss of RGCs in glaucoma was provided by the observations that microglial cells proliferate in the vicinity of RGCs [109] and that the recruitment and activation of microglial cells occurs before RGC death [99]. Additionally, minocycline, a tetracycline derivative known to inhibit microglial activation [110], suppresses RGC neurodegeneration in ischemia and glaucoma models [111–113] and improves the integrity of the optic nerve [104], further supporting a role for microglia to glaucomatous neuropathy. Furthermore, a high-dose of irradiation has been shown to reduce microglia reactivity and proliferation in the central retina and in the ONH region of animal models of glaucoma [104]. The reduction of microglia reactivity is associated with decrease in RGC degeneration and an improvement of the structural and functional integrity of RGC axons [104].

In eyes from glaucomatous patients, microglial cells present a more amoeboid morphology, clustering in the lamina cribosa and surrounding blood vessels, suggesting a protective role against damage to the blood-retinal barrier (BRB) [114]. In animal models of ocular hypertension [100] and chronic glaucoma [99], microglial cells become reactive and redistribute in the retina, optic nerve, and optic tract as early alterations, which may contribute to the disease onset or progression. Furthermore, in animal models of chronic glaucoma, the number of microglial cells double from 4 to 10 months in a reactive, not proliferative, gliosis response [109]. Additionally, in glaucomatous animal models, increased expression of major histocompatibility complex II (MHC-II) and CD200 (markers of activated microglia) is early detected in the retina, namely, adjacent to the optic nerve, suggesting this process accompanies ongoing axonal degeneration [97, 100, 115–118].

Reactive microglial cells are also observed in all retinal layers of eyes contralateral to experimental glaucoma, though with different morphology, suggesting an attempt of maintaining tissue homeostasis, protecting axons of the noninjured eye [116, 117, 119].

Microglia reactivity in glaucoma is not confined to the retina. Increased microglia reactivity following ocular hypertension is also apparent in the optic nerve and optic tract [100]. Activated microglial cells in the LGN, the primary processing center for visual information received from the retina, have also been observed in glaucomatous monkeys* in vivo* with positron emission tomography imaging with [^11^C]PK11195 [120, 121]. Neuronal degeneration in the LGN has been reported to occur in experimental primate and human glaucoma [121–125], which can be correlated with microglia activation [121].

### 4.2. Age-Related Macular Degeneration

Age-related macular degeneration is a degenerative disease that affects RPE and photoreceptors in the human macula. Early stages of the disease feature deposition of extracellular debris, known as drusen, from the basal side of the RPE into Bruch's membrane (thin stratified extracellular matrix that separates RPE from the choriocapillaris). The disease may progress into two forms: a slowly developing geographic atrophy (GA) form, also known as dry AMD, and the fast developing neovascular AMD (nAMD), also known as wet form or exudative form (reviewed in [126, 127]). Patients with GA exhibit areolar loss of the photoreceptors and RPE in the macula, whereas patients with nAMD exhibit angiogenesis and edema, from choroidal vessels that disrupt the overlying structures, including Bruch's membrane, RPE, and photoreceptor cells, resulting in focal retinal detachment and vision loss [126, 128].

No effective treatment or cure is currently available to treat the majority of AMD patients. Increased levels of vascular endothelial growth factor (VEGF) are detected in AMD patients, which can promote the exacerbation of choroidal neovascularization [129]. In fact, in spite of the use of anti-VEGF therapy there is a persistent activity of neovascular lesions [130].

AMD is a multifactorial disease with numerous risk factors associated (age, smoking status, obesity, and dietary fat consumption) [127, 131] but it also has a genetic predisposition to its development [132, 133]. Genome-wide association studies (GWAS) successively identified common risk variants localized in several candidate genes that are potentially involved in the development and progression of the disease [134–136]. Most of these genes are implicated in inflammatory pathway, implicating the immune system and inflammatory responses in the development and progression of AMD [137–139]. The imbalance between parainflammation and chronic inflammation leads to tissue damage and contributes to the initiation of AMD (reviewed in [140]). The intravitreal administration of corticosteroids, which are commonly used to treat inflammatory eye diseases, decreases the expression of presenting antigens from the MHC-II of microglia in AMD patients [141]. Indeed, the levels of TNF are increased in AMD patients, which suggested anti-TNF as an effective tool in AMD treatment [142, 143]. However, some studies demonstrated that some patients develop intraocular inflammatory reaction after intravitreal injection of infliximab [144, 145] and it has been shown that this treatment can be toxic depending on the dose administered [146].

In the laser photocoagulation animal model, known to induce choroidal neovascularization [147], the increased productions of intracellular adhesion molecule 1 (ICAM-1) and IL-6 were prevented by administration of astaxanthin, known by its antioxidative and anti-inflammatory properties [148], reinforcing the role of the inflammatory response in the disease.

Recruitment of microglia has been associated with progression and severity of AMD. In mouse models of AMD, the release of VEGF by monocytes and microglia, recruited to the subretinal space, plays a crucial role in choroidal blood vessel growth [149, 150]. In fact, blocking VEGF receptor inhibited the infiltration of microglia and macrophages in the laser photocoagulation animal model [151].

The strongest risk factor for the development of AMD is advanced age [152]. Interestingly, aged microglial cells present a reactive phenotype, which includes changes in morphology and surveillance impairment and may be correlated with AMD onset [153, 154]. In fact, similarly to what occurs in aged mice [155], accumulation of microglia in the subretinal space has been reported in animal models of AMD [156, 157]. Moreover, drusen accumulation attracts macrophages during the initial phases of AMD [158]. Activated microglial cells are also detected in ONL of AMD patients, which has been proposed to contribute to photoreceptor degeneration [149, 159, 160]. Spectral domain optical coherence tomography (SD-OCT) is a valuable tool for the* in vivo* evaluation of single retinal layers (both the inner retina and the outer retina), which has been used for the evaluation of hyperreflective retinal spots. Recently, in wet AMD patients, hyperreflective dots were reported as small-sized punctiform hyperreflective elements, scattered throughout all retina layers but mainly located in the outer retina layers around fluid accumulation, consistent with activated microglia [161].

The association of polymorphisms in the CX3CR1 gene with AMD [149, 162, 163] provided additional evidence for the contribution of microglia to the onset and development of AMD, since in the retina this gene is present only in microglia [164]. In CX3CR1-deficient mice, accumulation of microglia and macrophages in the subretinal space has been observed, contributing to drusen formation and photoreceptors degeneration [149, 165]. Similarly, it has been proposed that mutations in the CX3CR1 gene induce recruitment of monocytes/microglia into the subretinal space in the eyes of patients with AMD [163]. Therefore, CX3CR1-dependent regulation of microglia recruitment in the subretinal space appears to be involved in the development of both wet and dry AMD [149, 150].

The complement system is a component of the innate immune response that provides a rapid defense against a range of immunological challenges and contributes to the maintenance of homeostasis. Although activation of the complement system has beneficial properties including promoting the clearance of debris, immune complexes, and apoptotic cells, it may also exacerbate degeneration if activated in an inappropriate manner [166]. Age-related alterations in gene expression associated with the complement system, namely, component complement 3 (C3), complement factor B (CFB), and complement factor H (CFH), have already been described, being also associated with the AMD onset [167]. Deregulation of the complement system is considered to be one of the major factors contributing to the etiology of AMD [168]. Component complement 3 is a key component of the complement system and adenovirus-mediated delivery of C3 to murine RPE induces significant functional and anatomic changes that reproduce many of the features of AMD [168]. Genome-wide association studies have found an association between C3 allele variant and a high risk for AMD [169–171]. In addition, retinal microglial cells were recently identified as the cell type responsible for the synthesis and deposit of C3 in the outer retina during damage [172] or aging [173], reinforcing their role in the development of AMD. Moreover, accumulation of A2E, a bisretinoid constituent of ocular lipofuscin, which accumulates in an aged-dependent manner in microglia present in the outer retina, was recently reported to favor the activation of the complement system [174].

### 4.3. Diabetic Retinopathy

Diabetes mellitus is a group of metabolic diseases characterized by elevated blood glucose levels (hyperglycemia). Two broad categories encompass the majority of diabetes: type 1 diabetes results from the inability to produce and secrete insulin and type 2 diabetes is characterized by a chronic insulin resistance which may be accompanied with a relative deficiency in insulin secretion. The resulting chronic hyperglycemia that occurs in both types of diabetes is associated with long-term damage, dysfunction, and failure of various organs, especially the kidneys, nerves, heart, blood vessels, and the eye [175].

Diabetic retinopathy is one of the most common complications of diabetes mellitus and the most frequent cause of new cases of blindness among adults aged 20–74 years [176]. After 20 years of diabetes, nearly all patients with type 1 and more than 60% of the patients with type 2 diabetes have some degree of retinopathy [176].

Clinically, diabetic retinopathy has been considered a microvascular disease, characterized by increased vascular permeability, due to the breakdown of BRB [177]. However, retinal neurons are also affected by diabetes [178]. In fact, we and others demonstrated that diabetic conditions induce neural cell death in retinal cultures [179], in streptozotocin- (STZ-) induced type 1 diabetes rat model [180] and in diabetic patients [181]. Moreover, alterations in electroretinograms (ERG), which measure the electrical activity of the retina in response to light stimulus, were reported in diabetic patients [182–184] and in animal models of diabetes [185, 186]. These changes precede vascular alterations, suggesting that diabetic retinopathy should be classified as neurovascular disease [178], including a neurodegenerative component [184, 187].

Similar to other neurodegenerative diseases, diabetic retinopathy exhibits characteristics of low-grade chronic inflammation [188], with changes in retinal expression of inflammatory transcripts, which occurs in concert with functional changes in retinal permeability and apoptosis [189]. Proinflammatory cytokines, as TNF and IL-1*β*, were found to be increased in the vitreous of patients with diabetic retinopathy [190–192], as well as IL-6 [193]. Among numerous effects promoted by TNF in the CNS, particularly in the retina, several are intimately related to alterations observed in diabetic retinopathy, such as increased endothelial cell permeability [194], breakdown of BRB [195], and induction of leukocyte adhesion [196]. Blockade of the TNF pathway with antibodies against TNF receptor 1 (TNFR1), one of the TNF receptors associated with cell death [197], prevented not only the retinal vascular alterations of diabetes [198–201], but also the death of retinal neurons induced by elevated glucose concentration [202]. IL-1*β*, another proinflammatory cytokine, is also increased in the retina in experimental diabetes [203–205]. Interleukin converting enzyme/caspase-1, the enzyme responsible for the production of biological active IL-1*β* [206], is activated in the retina in both human and experimental diabetic retinopathy [207, 208]. In diabetic rat retinas, the increase in IL-1*β* levels is correlated with an increase in BRB permeability [209] and treatment with cyclosporine A, an anti-inflammatory drug, decreased both IL-1*β* levels and vascular permeability [210]. Experimental studies have also showed that intravitreal administration of IL-1*β* increases vascular permeability, which appears to be mediated by leukocyte adhesion, nuclear factor kappa-B activation, and retinal capillary cell death [204, 211], suggesting that this inflammatory cytokine might also play an important role in the pathogenesis of diabetic retinopathy. Nitric oxide has been shown to play a role in diabetic retinopathy pathology. In fact, in diabetic retinas there is an upregulation of iNOS and endothelial nitric oxide synthase (eNOS) [212–214]. Increased NO levels may contribute to BRB breakdown, since increased BRB permeability was not observed in the retinas of diabetic iNOS knockout mice [214, 215].

Accumulation of microglia in subretinal space was observed in a rat model of spontaneous type 2 diabetes, where the pores formed in RPE cells were a migratory pathway for inflammatory cells (microglia/macrophages) [216]. Moreover, microglial cells releasing proinflammatory mediators have been detected in the retina of diabetic animals [203, 217–219] and in human diabetic patients [220], as early as electroretinographic modifications [221]. Recently, SD-OCT documented discrete hyperreflective spots, which may correspond to aggregates of activated microglia that increased with the clinical progression of diabetic retinopathy [222].

Taking the role of microglia in diabetic retinopathy, pharmacologic regulation of microglia activity has been tested as a rational approach to modulate early pathological events associated with diabetic retinopathy. Genistein, which is an isoflavonoid naturally occurring and a tyrosine kinase inhibitor, has been demonstrated to inhibit retinal microglia activation in diabetes-induced inflammation, through inhibition of ERK and p38 phosphorylation [223]. Minocycline, a second-generation, semisynthetic tetracycline with anti-inflammatory properties, inhibits activation of retinal microglia and proinflammatory cytokines expression in experimental diabetes [203].

Recently, some clinical trials have investigated whether the inhibition of microglia could be a therapeutic strategy for the treatment of diabetic retinopathy. In a pilot proof-of-concept study (single-center, prospective, and open-label phase I/II clinical trial) with patients with diabetic macular edema, the treatment with minocycline improved visual function, central macular edema, and vascular leakage [224]. In a randomized, double-masked clinical trial in patients with severe nonproliferative diabetic retinopathy or non-high-risk proliferative diabetic retinopathy, doxycycline, a semisynthetic second-generation tetracycline also with anti-inflammatory properties, improved foveal frequency doubling perimetry, suggesting a link between a low-dose of this oral anti-inflammatory agent and subclinical improvement in inner retinal function [225].

The role of microglia reactivity in diabetic retinopathy remains unclear and a better understanding of this process could improve the development of new therapeutic strategies.

## 5. Conclusions

**Figure 3:**
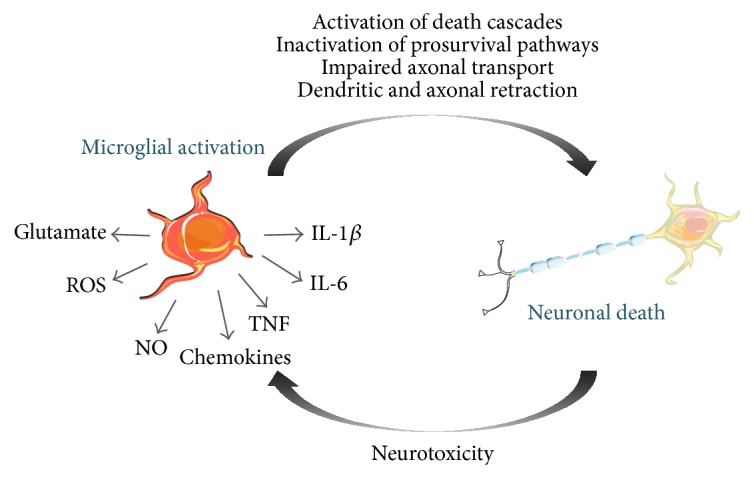
Relationships between microglial activation and neuronal cell death. In response to changes in the environment, microglia change to a more reactive phenotype, characterized by alterations in cell morphology, gene expression and proinflammatory mediators release. The sustained release of inflammatory factors perpetuates the neuroinflammatory process further activating microglia, which release proinflammatory and neurotoxic factors, contributing to neuronal dysfunction and to pathology.

There is mounting evidence demonstrating that microglia-mediated neuroinflammation plays an important role in the pathophysiology of several retinal degenerative diseases, but it remains to clarify whether microglia reactivity is the cause or consequence of the neurodegenerative process (Figure 3).

The contribution of other cells cannot be discarded, since infiltration of immune circulating cells, like macrophages, or other cells like astrocytes or Müller cells may also participate in the inflammatory process in retinal diseases.

Therapeutic strategies designed for reducing inflammation may offer beneficial effects for the management of retinal degenerative diseases. More preclinical and clinical studies are definitely needed to clarify the role of microglia in the onset and progression of retinal neurodegenerative diseases. The complete inhibition of microglia will have to be taken into account in further studies, taking into consideration the crucial role of microglia in homeostasis.
